# Supplementary figures and images for: Squalene in oil-based adjuvant improves the immunogenicity of SARS-CoV-2 RBD and confirms safety in animal models
Source: PLoS One. 2022 Aug 23;17(8):e0269823. doi: 10.1371/journal.pone.0269823 (PMC9397949; doi:10.1371/journal.pone.0269823)

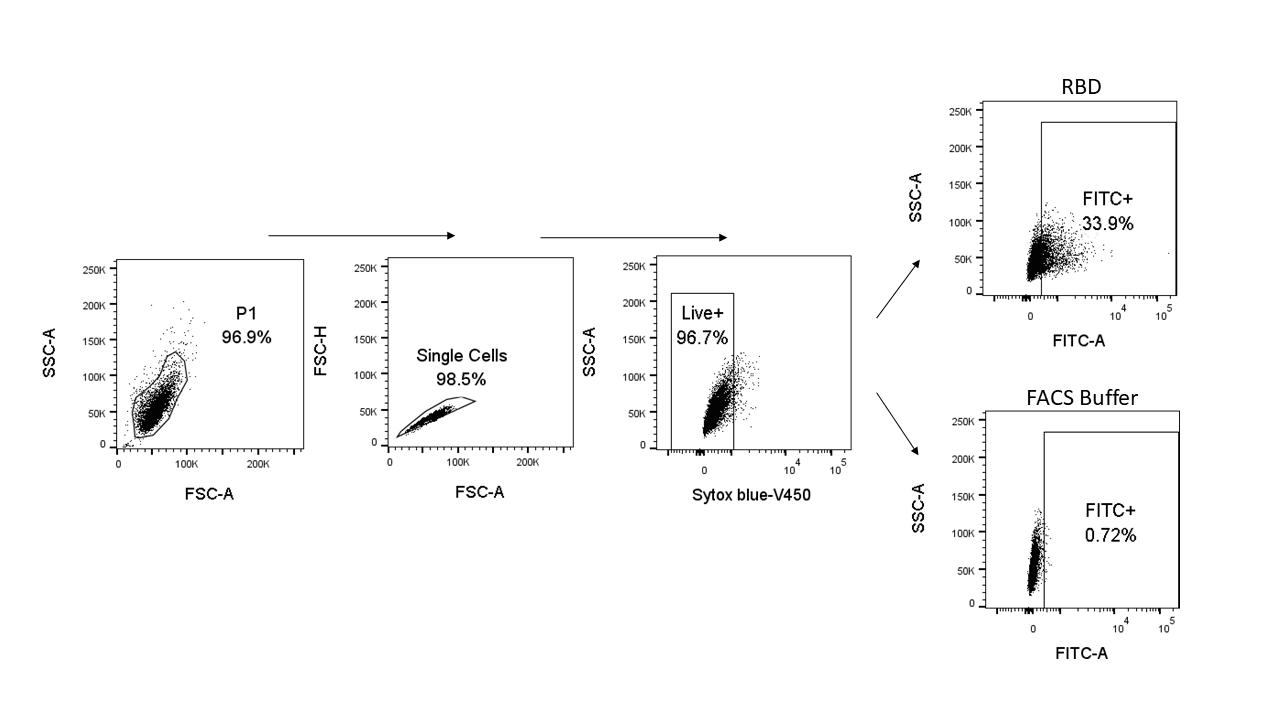

Supplement: S1 Fig — (TIF) [file pone.0269823.s001.tif]

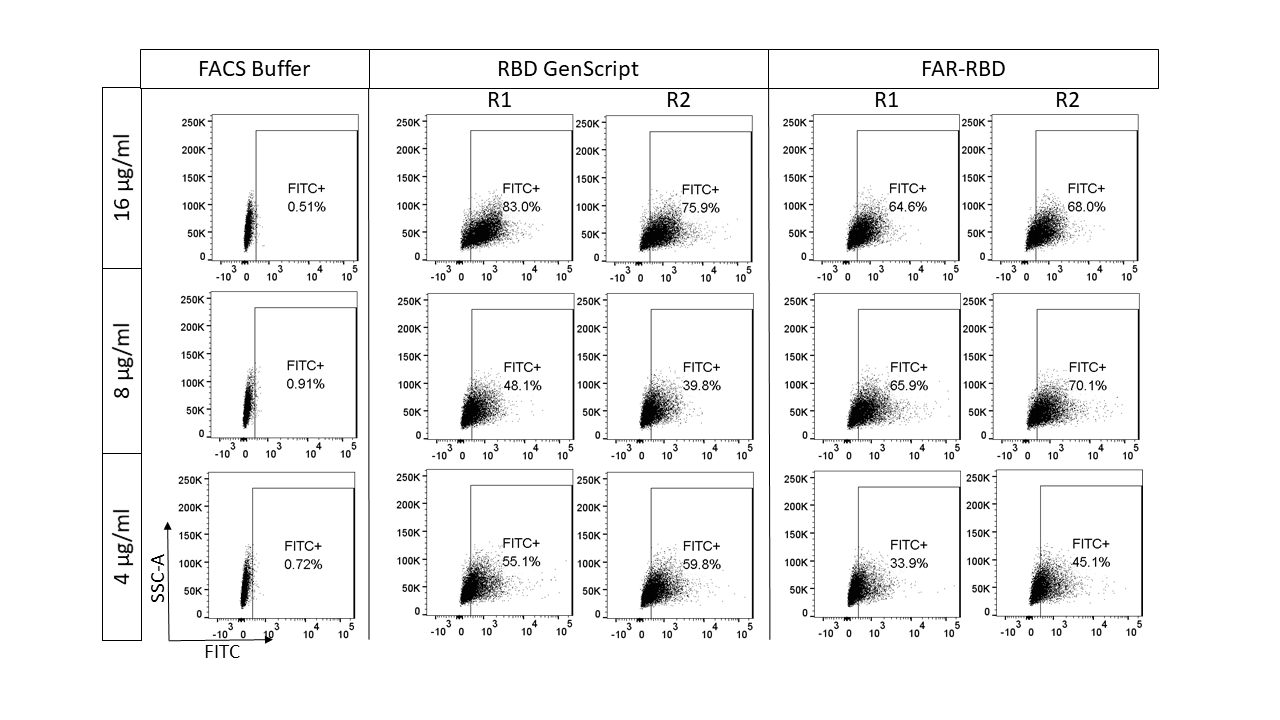

Supplement: S2 Fig — Two replicates were performed for each RBD evaluated. (TIF) [file pone.0269823.s002.tif]

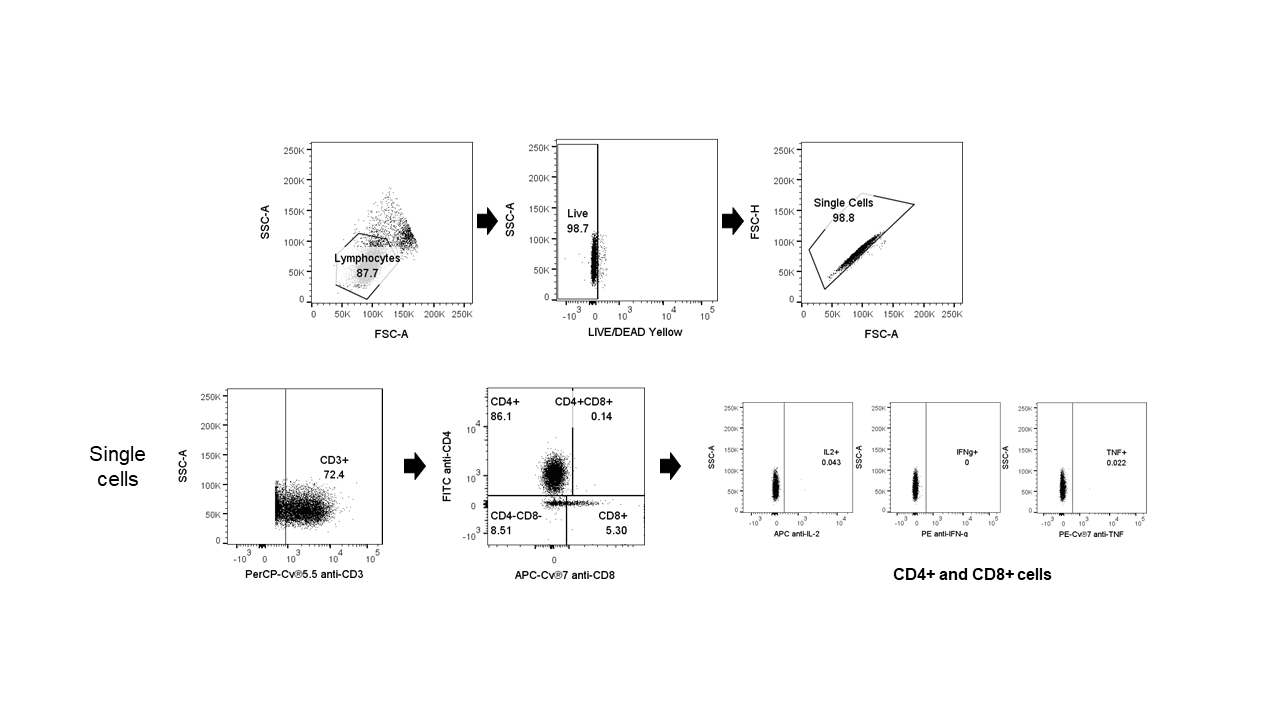

Supplement: S3 Fig — (TIF) [file pone.0269823.s003.tif]

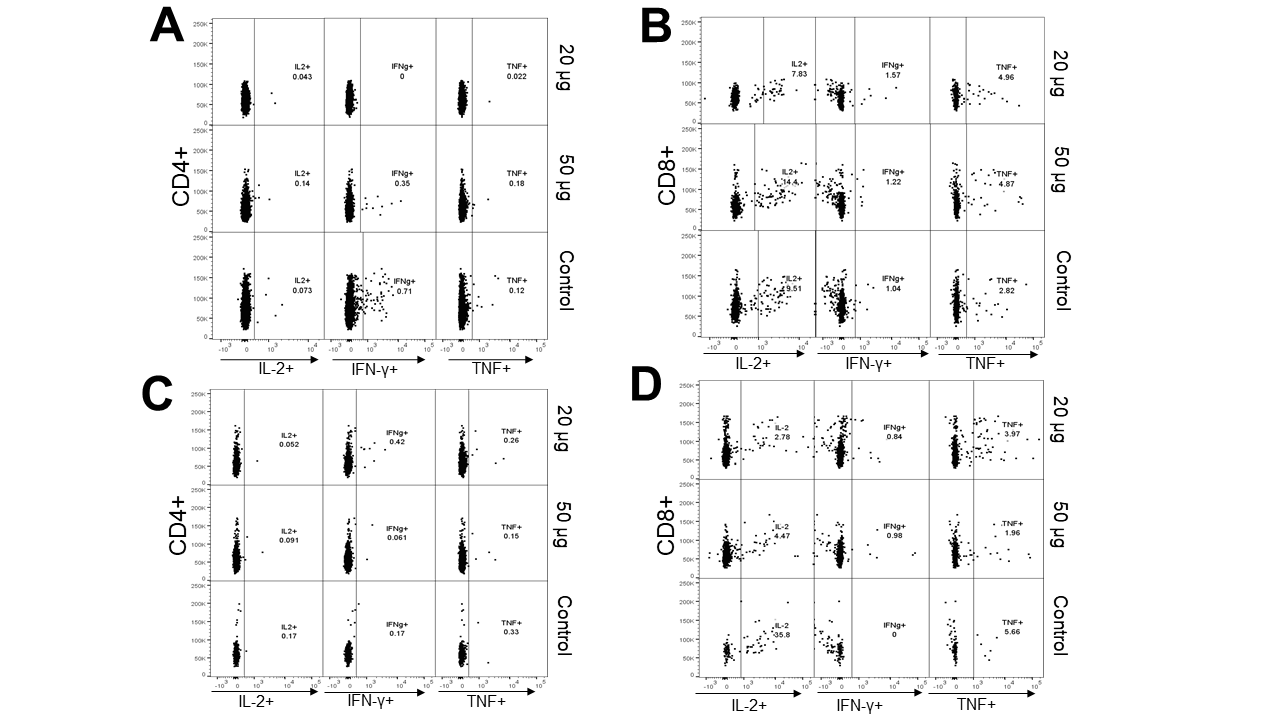

Supplement: S4 Fig — Groups immunized with adjuvant 1 (A, B). Groups immunized with adjuvant 3 (C, D). (TIF) [file pone.0269823.s004.tif]
