# Supplementary material for: Squalene in oil-based adjuvant improves the immunogenicity of SARS-CoV-2 RBD and confirms safety in animal models
Source: PLoS One. 2022 Aug 23;17(8):e0269823. doi: 10.1371/journal.pone.0269823 (PMC9397949; doi:10.1371/journal.pone.0269823)
Supplement: S1 Raw images — (PDF) [file pone.0269823.s006.pdf]

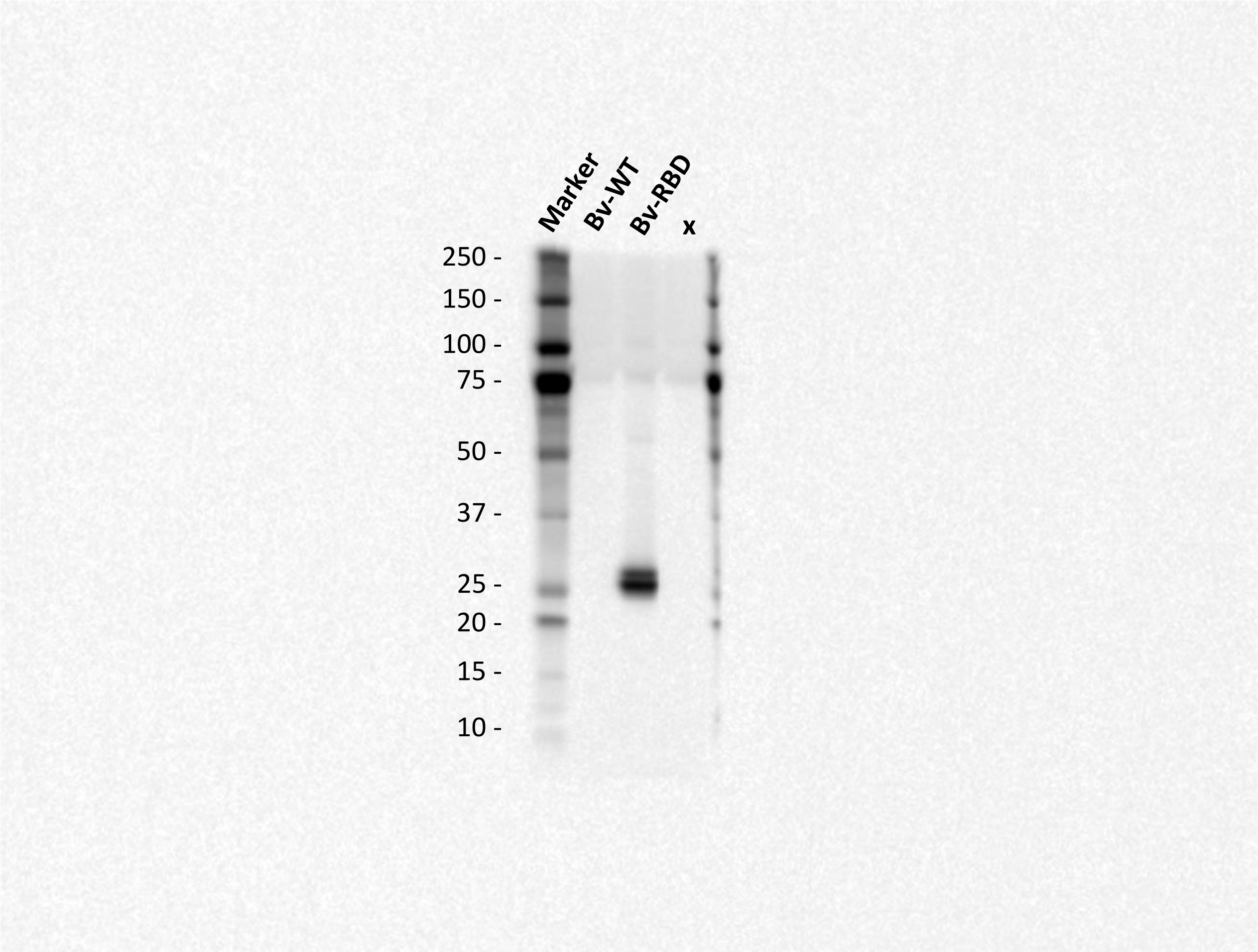

This blot corresponds to the figure 3B, detection of RBD using an anti His antibody. The image was obtained with a CCD camera in an Azure C600 Imaging system.

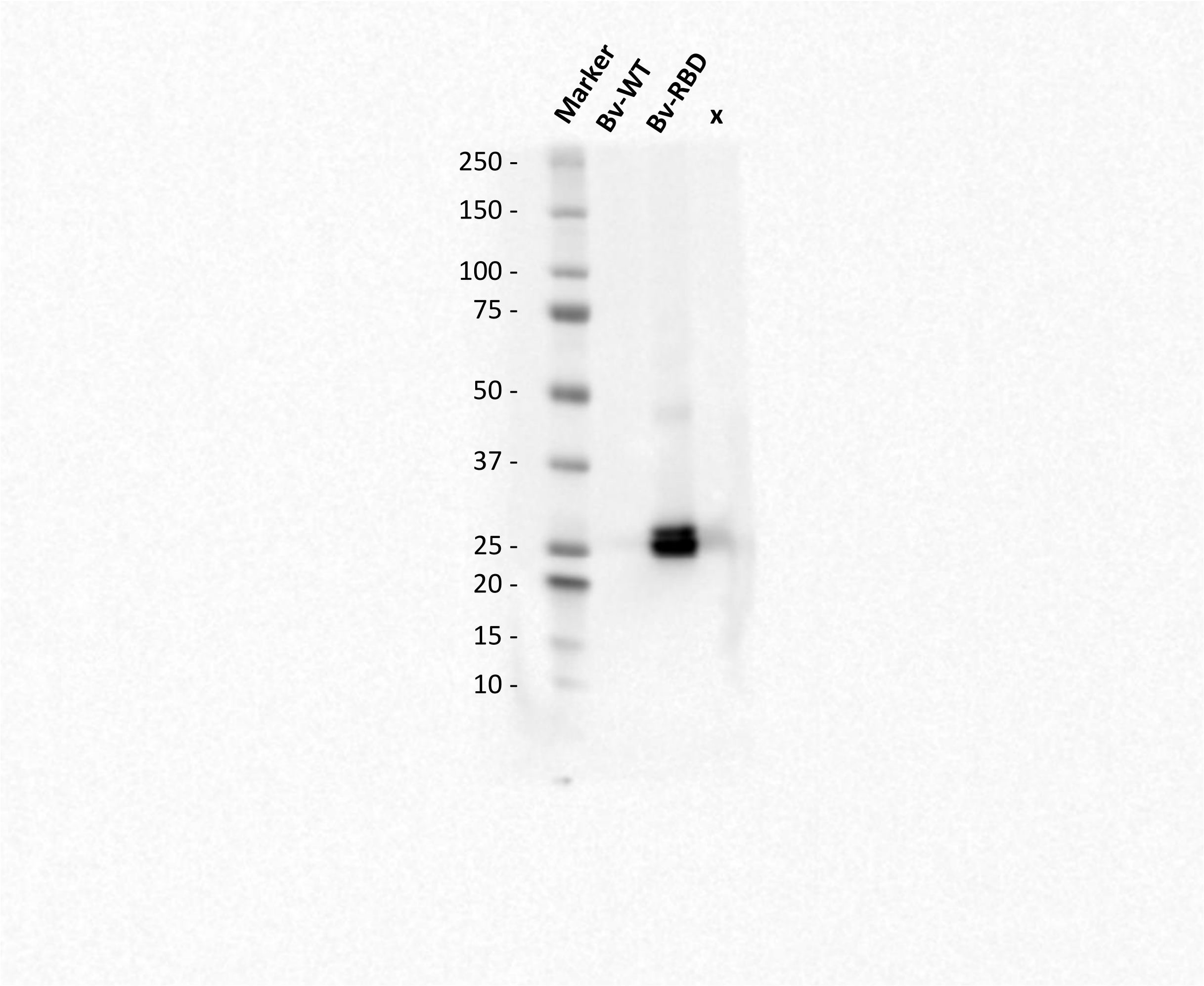

This blot corresponds to the figure 3B, detection of RBD using a polyclonal anti-spike antibody. The image was obtained with a CCD camera in an Azure C600 Imaging system.

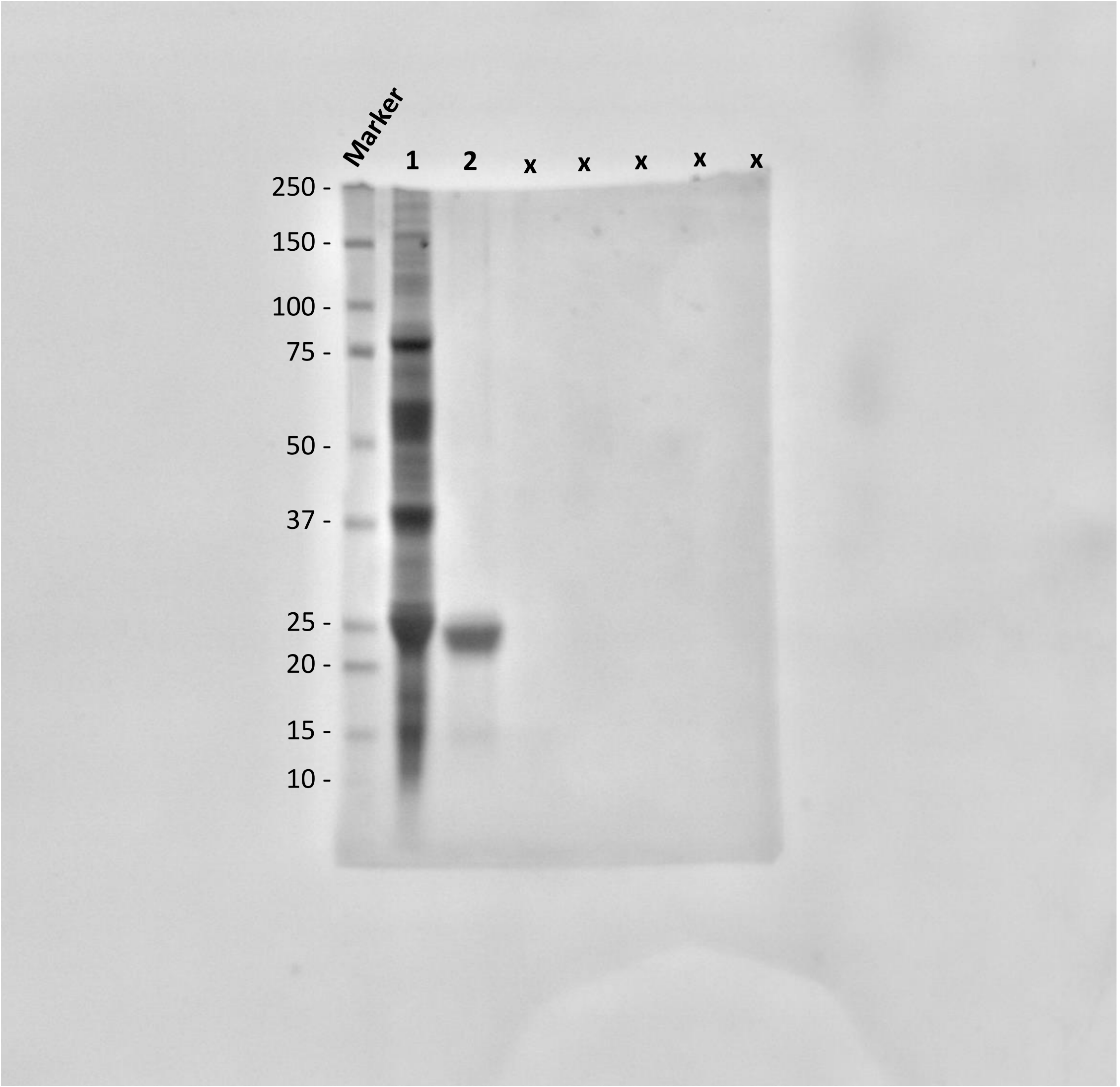

Lane 1: RBD after affinity chromatography purification step

Lane 2: RBD after size exclusion purification step

This gel corresponds to the figure 3C, Coomassie staining of the RBD obtained in each purification step. The image was obtained with a CCD camera in an Azure C600 Imaging system.

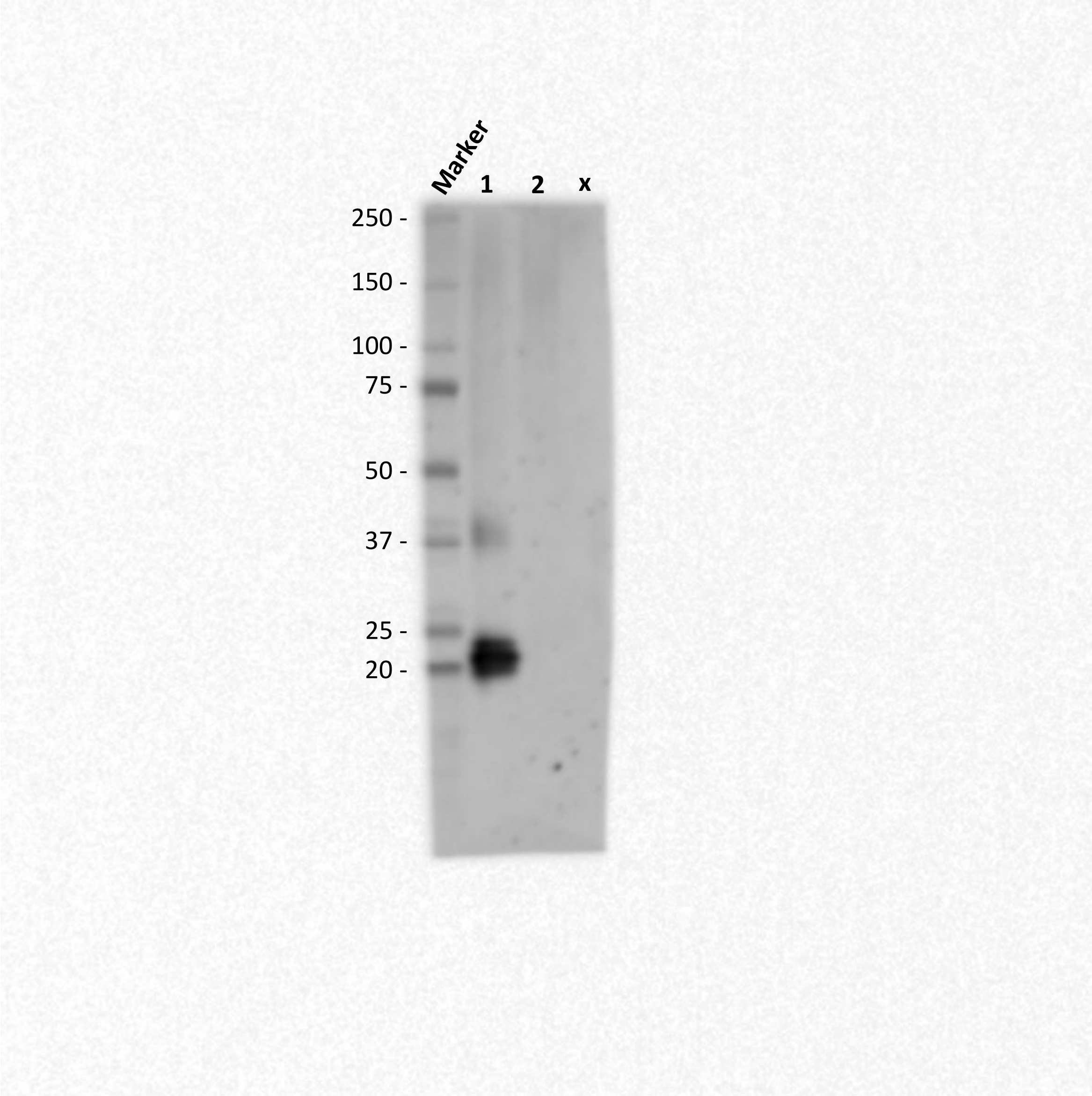

Lane 1: RBD in non-reducing conditions

Lane 2: RBD in reducing conditions

This blot corresponds to the figure 4B, detection of RBD in reducing and non-reducing conditions using sera of hamsters immunized with a NewCastle Disease virus (NDV) expressing the S1 of SARS-CoV-2. The image was obtained with a CCD camera in an Azure C600 Imaging system.
