# Supplementary material for: Squalene in oil-based adjuvant improves the immunogenicity of SARS-CoV-2 RBD and confirms safety in animal models
Source: PLoS One. 2022 Aug 23;17(8):e0269823. doi: 10.1371/journal.pone.0269823 (PMC9397949; doi:10.1371/journal.pone.0269823)
Supplement: Attachment — Submitted filename: Response to reviewers.pdf [file pone.0269823.s008.pdf]

Rebuttal Letter:

REF: PONE-D-21-36362R1

Squalene in oil-based adjuvant improves the immunogenicity of SARS-CoV-2 RBD and confirms safety in animal models

Dear Dr. Paulo Lee Ho, Ph.D.
Academic Editor
PLOS ONE

Please find below, our responsens and corrections to the reviewer´s comments:

Review Comments to the Author
if it exceeds 20,000 characters)

-Reviewer #2: I have reviewed the manuscript "Squalene in oil-based adjuvant improves the immunogenicity of SARS-CoV-2 RBD and confirms safety in animal models" which i previously reviewed in its first version and I agree to be published in its current version. The authors have responded to all comments convincingly and have also made all the required modifications. My best regards

Response:

We thank Dr. Reda Salem for the comments that made possible to improve our manuscript.

-Reviewer #3: This is an interesting article on the use of RBD produced in insect cells and formulated in emulsions with squalene as a potential vaccine against covid-19.

The questions raised by the reviewers seem to me to have been adequately answered by the authors. However, there are two additional small issues that I would like to point out: line 121 mentions the use of a tail with 10 histidine residues in the construction of the RBD, however, in figure 3, the scheme indicates 6 residues. Please correct.
Also, please review the symbol for kilo (lowercase k) throughout the text.

Response:

We are grateful with the reviewer for detecting these typos. All of them have been corrected (line 121) throughout the text.
